# Prevalence of diarrhoea, acute respiratory infections, and malaria over time (1995-2017): A regional analysis of 23 countries in West and Central Africa

**DOI:** 10.7189/jogh.11.13008

**Published:** 2021-08-10

**Authors:** Aline Simen-Kapeu, Lisa Bogler, Ann-Charline Weber, John Ntambi, Noel Marie Zagre, Sebastian Vollmer, Rene Ehounou Ekpini

**Affiliations:** 1 United Nations Children’s Fund (UNICEF), West and Central Africa Regional Office, Dakar, Senegal; 2 Department of Economics and Centre for Modern Indian Studies, University of Göttingen, Göttingen, Germany; 3 UNICEF Area Representative for Gabon and São Tomé and Príncipe and to the ECCAS, Libreville, Gabon

## Abstract

**Backgound:**

The global community recognizes the urgent need to end preventable child deaths, making it an essential part of the third Sustainable Development Goal. Pneumonia, diarrhoea, and malaria still remain the leading causes of deaths among children under five years, especially in one of the poorest geographic regions of the world – West and Central Africa. This region carries a disproportionately high share of the global burden, both in terms of morbidity and mortality. The study aims to assess levels and trends of the prevalence of these three childhood diseases in West and Central Africa to better inform ongoing and future programmes to improve child survival.

**Methods:**

Demographic and Health Surveys and Multiple Indicator Cluster Surveys available from 1995 to 2017 for 23 countries in West and Central Africa were analysed. We estimated the prevalence of diarrhoea, acute respiratory infections (ARI), malaria, and fever as a proxy for malaria, and split the data into three time periods to assess these trends in disease prevalence over time. Further analyses were done to assess the variations by geographic location (urban and rural) and gender (boys and girls).

**Results:**

In West and Central Africa, the reduction of the prevalence rates of diarrhoea, acute respiratory infections, malaria, and fever has decelerated over time (1995-2009), and little improvements occurred between 2010 and 2017. The reduction within the region has been uneven and the prevalence rates either increased or stagnated for diarrhoea (nine countries), ARI (four countries), and fever (six countries). The proportion of affected children was high in emergency or fragile settings. Disaggregated analyses of population-based data show persistent gaps between the prevalence of diseases by geographic location and gender, albeit not significant for the latter.

**Conclusions:**

Without intensified commitment to reducing the prevalence of pneumonia, malaria, and diarrhoea, many countries will not be able to meet the SDG goal to end preventable child deaths. Evidence-driven programmes that focus on improving equitable access to preventive health care information and services must be fostered, especially in complex emergency settings. This will be an opportunity to strengthen primary health care, including community health programmes, to achieve universal health coverage.

A high prevalence of preventable childhood diseases continues to burden many low- and middle-income countries [1,2]. Pneumonia, diarrhoea, and malaria still remain the leading causes of deaths among children; they were responsible for approximately one third of global deaths among children under five in 2018, totalling an estimated 1.6 million deaths in this age group [3,4]. This is shocking considering that many of these deaths are preventable by timely, low-cost and high-impact interventions [4].

The global community recognizes the urgent need to end preventable child deaths in all countries, making it an essential part of global child survival goals and strategies including the United Nations Global Strategy for Women’s, Children’s and Adolescents’ Health (2016-2030) [5] and the Sustainable Development Goals (SDGs) [6]. Global health initiatives that translated into the Global Action Plan for the Prevention of Pneumonia and Diarrhoea (GAPPD) and the WHO Global Malaria Programme (GMP) strive towards a reduction in morbidity and mortality due to the three childhood diseases [1,7]. In great part due to such programmes and large-scale interventions, child mortality has been falling over the past decades [3,4] and so has the prevalence of childhood diseases [1,2]. However, progress is unequally distributed and slowing down [1,4].

Many countries in sub-Saharan Africa, especially in West and Central Africa (WCA), missed the Millennium Development Goals and are lagging far behind the SDGs targets of no more than 25 deaths per 1000 livebirths among under-five children [8]. The WCA region is facing numerous challenges including a shift in demographics (birth and death rates and patterns), rapid urbanization, population displacements, violent extremism, armed conflicts, insecurity and the succession of disease outbreaks. In addition, children have an increased likelihood of being exposed to multiple deprivations including poor access to health services, malnutrition, lack of access to clean water and basic sanitation facilities, other infections such as HIV, and overcrowded conditions and environmental contaminants like indoor and outdoor air pollution [9,10]. Over two-thirds of the global burden of pneumonia and diarrhoea mortality occurs in just 15 countries, and five of them are located in WCA including Nigeria, the Democratic Republic of the Congo, Chad, Niger, and Côte d’Ivoire [2]. Similarly, malaria continues to be a major cause of death for children, taking the life of a child every two minutes despite large investments in its reduction and despite the fact that the proportion of infected children has halved in endemic areas of Africa since 2000 [11]. The Democratic Republic of the Congo and Nigeria accounted for 84 million (54%) of total malaria cases [1].

Monitoring trends in disease prevalence is important to inform ongoing and future programmes to combat these diseases. Many studies exist that estimate disease prevalence in individual countries at different points in time, including for malaria [12], pneumonia [13,14], and diarrhoea [15-17]. Meta-analyses review those single-country studies to estimate global trends in morbidity and mortality in single years [18-20] or over time [21]. We use nationally representative data from 23 countries in WCA to analyse levels and trends in childhood disease (pneumonia, diarrhoea, malaria) prevalence both for the entire region and separately for each country. The study focuses on morbidity instead of mortality as the disease prevalence captures the demand for prevention and treatment more accurately than the number of deaths. The study aims to inform governments and policy makers during programme development to accelerate child survival in sub-Saharan Africa.

## METHODS

### Data

In this paper, we combined two data sources, the Demographic and Health Surveys (DHS) and Multiple Indicator Cluster Surveys (MICS). The DHS is administered by ICF International. In seven rounds since 1984, the DHS Program has collected nationally representative data in low- and middle-income countries. MICS is a data collection initiative by UNICEF and has collected data on women and children in low- and middle-income countries since 1995. In both surveys, women in reproductive age, typically between 15 and 49, were asked for information on all children ever born to them. The surveys apply a multistage stratified sampling design for the within-country selection of households. For each country, regions were defined, within which the population was stratified into urban and rural. For each stratified area, enumeration areas were randomly drawn and denoted as primary sampling units (PSUs). Selection of PSUs was based on a probability proportional to size, in this case the number of households. Within each PSU, all households were listed from the most recent population census. Applying an equal probability of systematic sampling, a fixed number of households in each PSU was selected for an interview. Weights for the calculation of nationally representative statistics are provided with the survey data. For the analysis across countries, we re-scaled weights using each country’s female population aged 15 to 49 years in the last year of the respective time period. This ensures that small countries do not excessively influence the estimation of overall disease prevalence.

In this analysis, we included data from 1995 to 2017 for all countries in WCA for which data was available.

### Outcomes

We analysed trends of the three childhood diseases diarrhoea, acute respiratory infections (ARI) as proxy for pneumonia, malaria, and fever as a proxy for malaria. All diseases were coded as dummy variables that equal one if the child had the disease. A child was defined as having diarrhoea or fever if his/her guardian responded that the child suffered from diarrhoea or fever in the past 24 hours. Malaria was identified by a positive result of the rapid diagnostic test. A child was defined as having ARI if he/she had cough in the past 24 hours and experienced short, rapid breathing. A stricter definition would identify the disease as ARI only if the breathing difficulties were related to a problem in the chest. However, only more recent surveys included the question whether the child’s difficulty in breathing was due to a problem in the chest or a blocked nose, which would result in fewer observations for the trend analysis. We looked at ARI as a proxy for pneumonia, despite evidence that it is not a very accurate indicator [22,23]. Unfortunately, the DHS and MICS do not contain more accurate data on pneumonia such as diagnostic tests.

### Statistical analysis

In order to assess trends in disease prevalence over time, we split the data into three time periods. Time periods were defined such that each country had at least one survey in each time period, as far as possible. This resulted in the earliest period containing surveys between 1995 and 2001, the middle period containing surveys between 2002 and 2009, and the most recent time period containing surveys between 2010 and 2017. In the following, we refer to these time periods as the early, mid, and most recent time period. Since rapid tests for identifying malaria were only conducted in more recent surveys, malaria prevalence could only be shown for the recent time period.

First, we estimated the prevalence of each disease for all countries in the three time periods. We then repeated this exercise for rural and urban households separately, and for male and female children separately. To highlight heterogeneity between countries, we also calculated the prevalence of each disease separately for each country.

## RESULTS

Our final samples included 100 surveys from 23 countries with data on diarrhoea, 99 surveys from 23 countries with data on ARI, 20 surveys from 12 countries with data on malaria, and 102 surveys from 23 countries with data on fever. The number of observations varied greatly between surveys.

**Figure 1:**
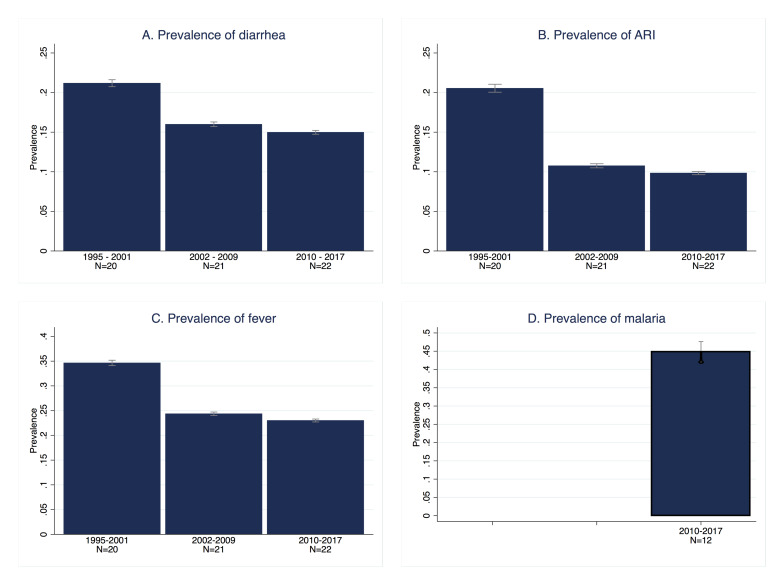
Prevalence of childhood diseases over time for all countries. **Panel A.** Prevalence of diarrhoea over time. **Panel B.** Prevalence of acute respiratory infections over time. **Panel C.** Prevalence of fever over time. **Panel D.** Prevalence of malaria.

**Figure 2:**
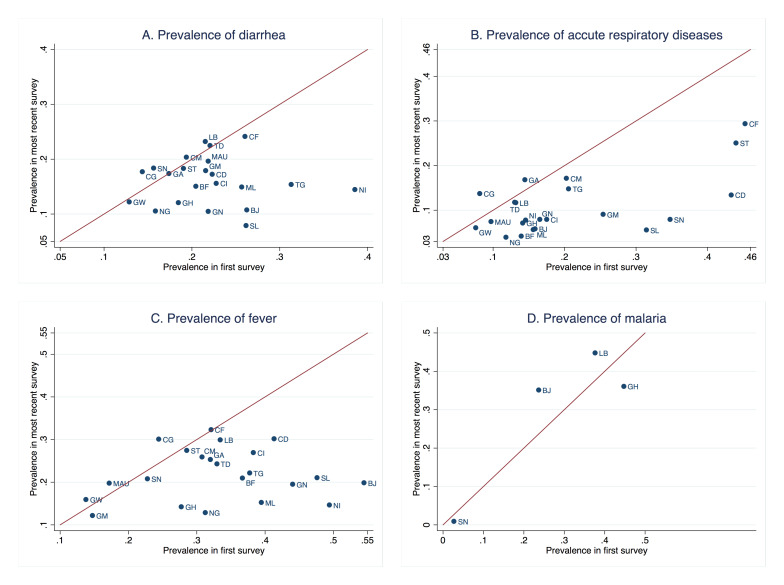
Change of prevalence of childhood diseases by country. **Panel A.** Change of prevalence of diarrhoea by country. **Panel B.** Change of prevalence of acute respiratory infections by country. **Panel C.** Change of prevalence of fever by country. **Panel D.** Change of prevalence of malaria by country.

**Figure 1****,** Panel A depicts the changes over time in the prevalence of diarrhoea for all included countries. As can be observed, the decline was substantial between the first two study periods, 1995-2001 and 2002-2009, but stagnated between the mid period and the most recent period (2010-2017). The pooled prevalence of diarrhoea first decreased from 21.2% to 16.0%, but remained at 15.0% in the most recent time period. **Table 1** shows the differences across countries. The latest estimates per country ranged from 24% in the Central African Republic (95% confidence interval (CI) = 23.0%-25.3%) and Equatorial Guinea (95% confidence interval (CI) = 21.7%-25.8%) to 8% in Sierra Leone (95% CI = 7.2%-8.6%). Prevalence remained rather high with the majority of countries exhibiting rates higher than 15% and only few countries with a lower prevalence, namely Sierra Leone, Guinea with 10% (95% CI = 9.2%-11.6%), Benin (95% CI = 10.0%-11.4%) and Nigeria (95% CI = 9.9%-11.2%) with 11%, Ghana (95% CI = 10.7%-13.4%) and Guinea-Bissau (95% CI = 10.8%-13.6%) with 12%, and Niger with 14% (95% CI = 13.5%-15.4%). Similarly, within-country reduction in the prevalence rates among the first and last available surveys showed great variation. Diarrhoea prevalence decreased in all countries except for Senegal, Cameroon and Liberia who faced increased rates, and Chad and Gabon where rates remained the same. In contrast, large reductions were observed in Niger between 1998 and 2012 (25 percentage points), Sierra Leone between 2000 and 2017 (18 percentage points), Togo between 1998 and 2013 (16 percentage points) and Benin between 1996 and 2017 (15 percentage points). However, the time spans between the first and most recent surveys varied between countries which increased the observed heterogeneity in trends. **Figure 2****,** Panel A presents the change in the prevalence of diarrhoea graphically. It illustrates the heterogeneity across countries as well as the trend of declining diarrhoea prevalence. In the **Online Supplementary Document**, figures for each country depict the trend in the prevalence of diarrhoea over time while taking into account the actual timing of each survey (Figure S5, similar for ARI and fever in Figure S6 and Figure S7).

**Table 1:** Prevalence of diarrhoea by country in first and most recent survey year

Country	Year	Prevalence	95% CI	Observations
Benin	1996	0.26	0.241-0.285	2721
	2017	0.11	0.100-0.115	12 154
Burkina Faso	1998	0.20	0.188-0.221	4922
	2010	0.15	0.141-0.159	13 425
Cameroon	1998	0.19	0.173-0.215	2054
	2014	0.20	0.190-0.217	6612
Central African Republic	2000	0.26	0.249-0.273	12 993
	2010	0.24	0.230-0.253	9994
Chad	1996	0.22	0.203-0.239	6135
	2014	0.22	0.212-0.238	16 405
Congo	2005	0.14	0.126-0.161	4158
	2014	0.18	0.166-0.188	8557
Congo Democratic Republic	2001	0.22	0.209-0.238	9838
	2013	0.17	0.159-0.185	16 535
Cote d‘Ivoire	1998	0.23	0.195-0.261	1605
	2016	0.16	0.145-0.167	8463
Equatorial Guinea	2000	0.24	0.217-0.258	2087
			
Gabon	2000	0.17	0.159-0.190	3655
	2012	0.17	0.157-0.189	5145
Gambia	2000	0.22	0.199-0.233	3357
	2013	0.18	0.160-0.198	7461
Ghana	1998	0.18	0.168-0.201	2927
	2014	0.12	0.107-0.134	5406
Guinea	1999	0.22	0.205-0.232	4814
	2016	0.10	0.092-0.116	6823
Guinea-Bissau	2006	0.13	0.117-0.141	5061
	2014	0.12	0.108-0.136	7053
Liberia	2006	0.22	0.195-0.236	4740
	2013	0.23	0.214-0.250	6462
Mali	1995	0.26	0.241-0.273	5123
	2015	0.15	0.138-0.159	15 233
Mauritania	2007	0.22	0.207-0.231	8064
	2015	0.20	0.182-0.210	9944
Niger	1998	0.39	0.365-0.407	4145
	2012	0.14	0.135-0.154	11 256
Nigeria	1999	0.16	0.144-0.173	3078
	2013	0.11	0.099-0.112	27 538
São Tomé and Príncipe	2000	0.19	0.170-0.211	1795
	2014	0.18	0.159-0.206	1905
Senegal	1997	0.16	0.145-0.168	6372
	2017	0.18	0.172-0.194	11 004
Sierra Leone	2000	0.26	0.242-0.281	2279
	2017	0.08	0.072-0.086	10 612
Togo	1998	0.31	0.292-0.334	3836
	2013	0.15	0.137-0.171	6358

CI – confidence interval

The overall trend of ARI among children is presented in **Figure 1****,** Panel B and **Figure 2****,** Panel B. The average prevalence in the region has decreased by half from 20.6% in the first period to 10.8% in the mid period and 9.8% in the most recent period. Most of the countries have experienced significant reductions apart from Congo and Gabon where the prevalence rates increased. **Table 2** presents prevalence rates of ARI at the country level for the first and most recent surveys. For most countries in the recent period the prevalence rates are below or equal to 15% while prevalence remained high in the Central African Republic with 29% (95% CI = 27.7%-31.0%), São Tomé and Príncipe with 25% (95% CI = 22.1%-28.0%) and Cameroon with 17% (95% CI = 15.6%-18.6%). The largest reductions between the first and most recent surveys were found in Democratic Republic of Congo between 2001 and 2013 (30 percentage points), Senegal between 2000 and 2017 (27 percentage points) and Sierra Leone between 2000 and 2017 (25 percentage points). In the case of Equatorial Guinea information was only available for the year 2000.

**Table 2:** Prevalence of ARI by country in first and most recent survey year

Country	Year	Prevalence	95% CI	Observations
Benin	1996	0.16	0.141-0.177	2711
	2017	0.06	0.052-0.064	12173
Burkina Faso	1998	0.14	0.124-0.155	4884
	2010	0.04	0.036-0.048	13 409
Cameroon	1998	0.20	0.179-0.227	2044
	2014	0.17	0.156-0.186	6607
Central African Republic	2000	0.45	0.432-0.474	7480
	2010	0.29	0.277-0.310	9972
Chad	1996	0.13	0.116-0.145	6116
	2014	0.12	0.105-0.129	16 318
Congo	2005	0.08	0.068-0.095	4064
	2014	0.14	0.125-0.149	8563
Congo Democratic Republic	2001	0.43	0.405-0.462	4862
	2013	0.13	0.118-0.150	16 512
Cote d‘Ivoire	1998	0.18	0.152-0.198	1597
	2016	0.08	0.068-0.089	8461
Equatorial Guinea	2000	0.36	0.329-0.393	1159
			
Gabon	2000	0.14	0.129-0.160	3648
	2012	0.17	0.147-0.188	5134
Gambia	2000	0.25	0.224-0.284	1460
	2013	0.09	0.077-0.104	7431
Ghana	1998	0.14	0.126-0.157	2920
	2014	0.07	0.061-0.081	5402
Guinea	1999	0.17	0.153-0.179	4803
	2016	0.08	0.070-0.088	6814
Guinea-Bissau	2006	0.08	0.065-0.086	5057
	2014	0.06	0.051-0.070	7046
Liberia	2006	0.13	0.116-0.148	4712
	2013	0.12	0.100-0.133	6453
Mali	1995	0.16	0.142-0.171	5048
	2015	0.06	0.049-0.063	15 238
Mauritania	2007	0.10	0.088-0.106	8016
	2015	0.07	0.064-0.084	9894
Niger	1998	0.15	0.130-0.161	4143
	2012	0.08	0.067-0.088	11 201
Nigeria	1999	0.12	0.106-0.131	3039
	2013	0.04	0.035-0.043	27 422
São Tomé and Príncipe	2000	0.44	0.396-0.484	712
	2014	0.25	0.221-0.280	1904
Senegal	2000	0.35	0.328-0.368	4313
	2017	0.08	0.071-0.087	11 005
Sierra Leone	2000	0.31	0.282-0.347	1410
	2017	0.06	0.049-0.062	10 648
Togo	1998	0.21	0.189-0.223	3806
	2013	0.15	0.132-0.163	6350

CI – confidence interval, ARI – acute respiratory infections

**Figure 1****,** Panel C depicts the change in the prevalence of fever for all included countries over time. Overall, fever prevalence decreased substantially. Rates declined from an average of 35.7% in the first study period (1995-2001) to 24.4% in the second period (2002-2009) and 23.0% in the last period (2010-2017). Variations across countries are described in detail in **Table 3**, depicting similar rates in the last available surveys for each country, but identifying different patterns in the reduction over time (**Figure 2****,** Panel C). Benin has achieved large improvements with a reduction of 35 percentage points in its prevalence rate between 1996 and 2017. Likewise, Sierra Leone (27 percentage points, 2000-2017), Guinea (25 percentage points, 1999 - 2016), Mauritania (24 percentage points, 2007-2015), Niger (24 percentage points, 1998-2012) and Nigeria (18 percentage points, 1999-2013) have shown a significant reduction across study periods. However, despite improvements, high levels of prevalence of fever persisted in some countries. This becomes clear when looking at prevalence levels in the Central African Republic with the highest rate of 32% (95% CI = 30.8%-33.7%), Congo (95% CI = 28.4%-31.7%), Liberia (95% CI = 27.5%-32.4%), and Democratic Republic of Congo (95% CI = 28.4%-31.9%) with rates of 30%, Sao Tome and Principe (95% CI = 24.7%-30.1%) and Cote d’Ivoire (95% CI = 25.2%-28.5%) with 27%, and Cameroon with a rate of 26% (95% CI = 24.4%-27.4%).

**Table 3:** Prevalence of fever by country in first and most recent survey year

Country	Year	Prevalence	95% CI	Observations
Benin	1996	0.55	0.523-0.568	2713
	2017	0.20	0.186-0.209	12 172
Burkina Faso	1998	0.37	0.347-0.387	4913
	2010	0.21	0.198-0.221	13 426
Cameroon	1998	0.31	0.278-0.338	2046
	2014	0.26	0.244-0.274	6616
Central African Republic	2000	0.32	0.309-0.333	13 030
	2010	0.32	0.308-0.337	9983
Chad	1996	0.33	0.306-0.353	6132
	2014	0.24	0.226-0.260	16 405
Congo	2005	0.24	0.224-0.265	4161
	2014	0.30	0.284-0.317	8561
Congo Democratic Republic	2001	0.41	0.392-0.434	9848
	2013	0.30	0.284-0.319	16 536
Cote d‘Ivoire	1998	0.38	0.351-0.416	1601
	2016	0.27	0.252-0.285	8466
Equatorial Guinea	2000	0.30	0.278-0.325	2080
			
Gabon	2000	0.32	0.299-0.341	3646
	2012	0.25	0.228-0.278	5147
Gambia	2000	0.15	0.126-0.169	3352
	2013	0.12	0.106-0.137	7454
Ghana	1998	0.28	0.258-0.298	2918
	2014	0.14	0.126-0.158	5406
Guinea	1999	0.44	0.422-0.459	4752
	2016	0.19	0.179-0.210	6824
Guinea-Bissau	2006	0.14	0.126-0.151	5066
	2014	0.16	0.148-0.170	7051
Liberia	2006	0.33	0.314-0.355	4725
	2013	0.30	0.275-0.324	6460
Mali	1995	0.39	0.375-0.414	5117
	2015	0.15	0.142-0.161	15 250
Mauritania	2007	0.17	0.159-0.185	8049
	2015	0.20	0.182-0.213	9917
Niger	1998	0.49	0.473-0.515	4145
	2012	0.15	0.135-0.157	11 245
Nigeria	1999	0.31	0.293-0.332	3078
	2013	0.13	0.121-0.136	27 491
São Tomé and Príncipe	2000	0.29	0.258-0.313	1792
	2014	0.27	0.247-0.301	1869
Senegal	2000	0.23	0.212-0.244	8121
	2017	0.21	0.195-0.221	11 005
Sierra Leone	2000	0.48	0.451-0.501	2278
	2017	0.21	0.199-0.222	10 632
Togo	1998	0.38	0.353-0.402	3816
	2013	0.22	0.203-0.239	6358

CI – confidence interval

The rapid test for malaria was implemented only in recent surveys and the information available covers a total of 12 countries (**Table 4**, **Figure 1****,** Panel D, **Figure 2****,** Panel D). **Table 4** shows malaria prevalence from the last available surveys at the country level. Burkina Faso had the highest prevalence rate of 76% (95% CI = 74.5%-77.6%) followed by Nigeria with 51% (95% CI = 46.4%-55.4%), Mali with 47% (95% CI = 43.4%-49.7)¸Guinea with 47% (95% CI = 42.8%-50.5%), Liberia with 45% (95% CI = 41.1%-48.5%) and Cote d'Ivoire with 41% (95% CI = 37.3%-44.3%). At the other extreme, Senegal and Gambia had a prevalence rate of 1 (95% CI = 0.5%-1.3%) and 2% (95% CI = 1.1%-3.4%), respectively.

**Table 4:** Prevalence of malaria by country in most recent survey year

	Year	Prevalence	95% CI	Observations
Benin	2017	0.35	0.328-0.374	5558
Burkina Faso	2010	0.76	0.745-0.777	5724
Congo Democratic Republic	2013	0.30	0.271-0.328	7276
Cote d‘Ivoire	2011	0.41	0.373-0.443	2864
Gambia	2013	0.02	0.011-0.034	2703
Ghana	2014	0.36	0.326-0.396	2373
Guinea	2012	0.47	0.428-0.505	2775
Liberia	2011	0.45	0.411-0.485	2426
Mali	2012	0.47	0.434-0.497	4182
Nigeria	2010	0.51	0.464-0.554	4352
Senegal	2017	0.01	0.005-0.013	9483
Togo	2013	0.37	0.338-0.406	2870

CI – confidence interval

In order to facilitate policy recommendations regarding targeting of health programmes, we disaggregated these overall prevalences by location (rural/urban) and gender. Graphs depicting the results are presented in Figures S1 to S4 in the **Online Supplementary Document**). Children in households located in rural areas had a higher prevalence of all childhood diseases analysed. The gap between the prevalence by location decreased only slightly since 1995. Girls had a lower prevalence of diarrhoea and to a lesser extent a lower prevalence of ARI and fever, although this difference is not statistically significant. The gap between the prevalence by gender neither decreased nor increased significantly since 1995.

## DISCUSSION

This study was conducted to assess levels and trends of the prevalence of childhood diseases during the past 25 years in WCA and with the aim of contributing to fill the literature gap between country-specific studies and global analyses of the prevalence and trends of those diseases. Our analysis found important progress in the reduction of the prevalence of diarrhoea, ARI, malaria, and fever in the pooled sample of countries between the periods 1995 and 2009. However, given the international attention placed on health in the SDGs, it is somewhat disheartening to see that relatively little progress was made at the aggregate level in the WCA region in the latest period 2010-2017.

These results highlight the proportion of the vulnerable populations, children under 5 years, affected by the three diseases in WCA, a particularly important region which includes countries with the highest prevalence rates worldwide, where these diseases are still an important contributor to the prevailing high levels of child mortality [3]. Prevalence rates continue to be high: 20% of children or more had diarrhoea in nine countries; at least 12% of children had symptoms of ARI in nine countries; the prevalence of fever was between 20% and 32% in 15 countries; and at least one third of children were tested positive for malaria in 10 countries. The three major killers of children under 5, pneumonia, malaria, and diarrhoea, can be addressed by highly effective and low cost preventive and therapeutic interventions, such as breastfeeding promotion, and *Haemophilus influenzae* type b and pneumococcal vaccines for pneumonia, improved water and sanitation, rotavirus vaccine, zinc supplementation, oral rehydration solutions, and integrated community case management of childhood illnesses, and insecticide treated bednets, intermittent preventive treatment in pregnancy, and artemisinin-based combination therapy for malaria [1,7].

The slowdown in the decline of prevalence rates might indicate diminishing returns of the traditional investments on health. Thus, it calls for more concerted efforts and the combination of different strategies to accelerate the progress in reducing the prevalence of childhood diseases. Weak health systems (including community health systems) in low-income countries pose a barrier to acquire adequate information, knowledge, and care, especially for the most vulnerable populations [24]. The need for appropriate culturally sensitive education and awareness provided to the communities to considerably reduce the number of children likely to get affected by the three diseases is more vital than ever before. Empowerment of women and removing barriers to access care may help inculcate healthy practices among children under 5, and their mothers, fathers, and caregivers [25]. Similarly, as parasite resistance to antimalarial medicines emerges, new treatments have to be developed and made available for affected populations.

Disaggregated analyses of population-based data show persistent gaps between the prevalence of diseases by location (urban and rural) and gender (boys and girls), albeit not significant for the latter. Protecting every child’s right requires addressing persistent inequities and disparities as no child should be left behind. As populations at risk grew, the average level of funding available per person at risk declined between 2012 and 2017 [1]. As jointly stated by all parties in the Astana Declaration in 2018 [26], considerable investments in strengthening primary health care, including community health programmes, is highly needed, to ensure that every child has access to preventive, promotional, and curative lifesaving interventions [5]. At the Global Forum on Pneumonia, organized by UNICEF, Save The Children, and partners in Barcelona in January 2020, all countries committed to accelerate progress by strengthening preventive health care services. Such initiatives are an important step and need to be translated into tangible actions.

The study further extends the literature by depicting within-region patterns that allow us to identify the fastest and slowest performing countries in the reduction of the prevalence of each specific disease. We noted an increase (Central African Republic, Gambia, Liberia, São Tomé and Príncipe, and Senegal) or no change (Chad, and Principe) in diarrhoea prevalence rates. ARI prevalence rates increased or stagnated in four countries (Chad, Congo, Gabon, and Liberia) and reduced in others. Fever prevalence rates reduced by at least 50% in seven countries but limited or no change in Central African Republic, Gambia, Liberia, São Tomé and Príncipe, and Senegal. While the progress made is likely to be driven to a large extent by major global initiatives, the country heterogeneities highlight that the same strategy does not work equally well in each context. This heterogeneity could be attributed to the country-specific context (including emergency settings) and differences in socio-economic and sociocultural practices. It could also be due to the methodological variation in the assessment of prevalence at the time of the survey.

Our study has several limitations. Information on diarrhoea, ARI, and fever is collected by asking the guardian about the child’s past diseases. This might not capture the disease accurately. Especially the definition of ARI does not serve as a very accurate indicator of pneumonia, which is a main cause of child mortality [22,23]. Unfortunately, DHS and MICS only contain medical tests for malaria and even this test is only conducted in selected surveys. Fever, while it is one symptom of malaria, is a rather inaccurate proxy for malaria, as it is a symptom of other diseases such as pneumonia as well. Data availability therefore greatly limits our analysis. Furthermore, the study is descriptive and does not provide an analysis of drivers of the trends in prevalence rates.

Despite these constraints, this study lays out potential areas of improvement in the current sources of information and data collection instances that will facilitate research on the progress towards the reduction of childhood illnesses. At the same time, it outlines potential areas of research, such as the identification of the factors that might explain the heterogeneity across countries in the region and the reasons behind the slowdown in the reduction of the prevalence rates of each disease observed between 2010 and 2017.

## CONCLUSION

Diarrhoea, acute respiratory infections, malaria, and fever are still highly prevalent in West and Central Africa. The reduction of these prevalence rates has decelerated over time (1995-2009), and little improvements occurred between 2010 and 2017. The large heterogeneity across countries and the over-representation of particular countries among those with the greatest prevalence rates in the region call for a renewed focus on high-burden countries and more analytical work to document and disseminate best practices of best-performing countries in terms of prevalence reduction. The speeding up in the achievement of global targets must be fostered, especially in complex emergency settings, by evidence-driven programmes that focus on improving access to preventive health care information and services. This will be an opportunity to strengthen primary health care to achieve universal health coverage. Without intensified commitment to reducing the prevalence of pneumonia, malaria, and diarrhoea, many countries will not be able to meet the SDG goal to end preventable child deaths.

## Additional material


Online Supplementary Document
