# Supplementary material for: Prevalence of diarrhoea, acute respiratory infections, and malaria over time (1995-2017): A regional analysis of 23 countries in West and Central Africa
Source: J Glob Health. 2021 Aug 10;11:13008. doi: 10.7189/jogh.11.13008 (PMC8397278; doi:10.7189/jogh.11.13008)
Supplement: Online Supplementary Document [file jogh-11-13008-s001.pdf]

Figures S1 – S4. Prevalence of childhood diseases over time, disaggregated by location and gender

Figure S1. Prevalence of diarrhoea over time, disaggregated by location and gender.

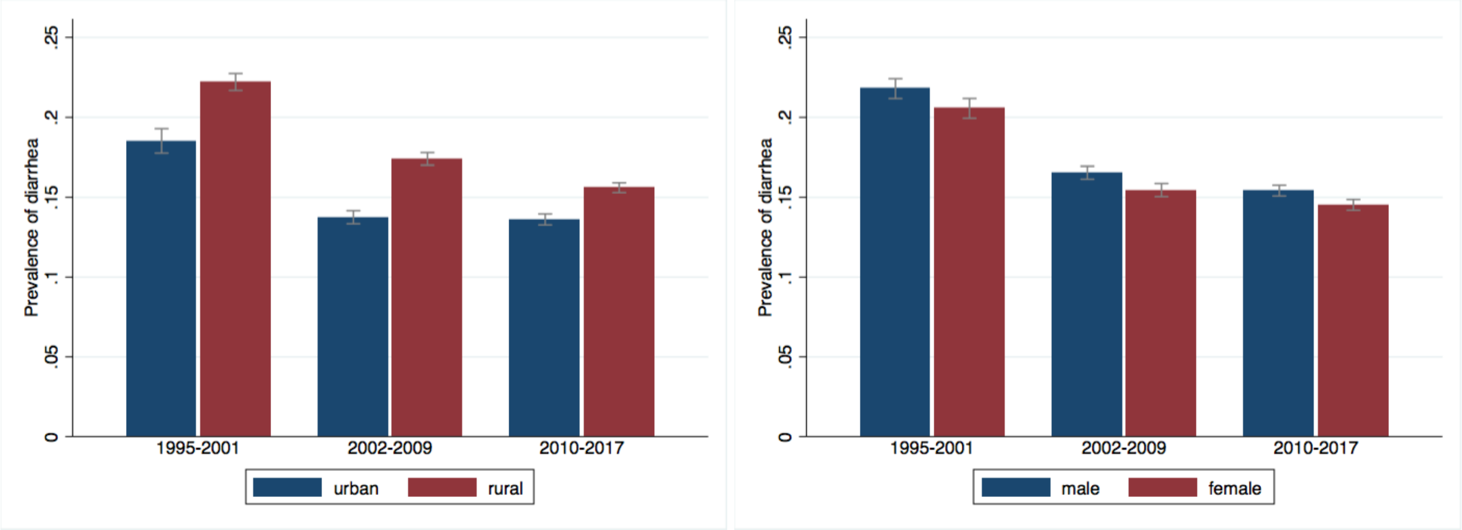

Figure S2. Prevalence of acute respiratory infections over time, disaggregated by location and gender.

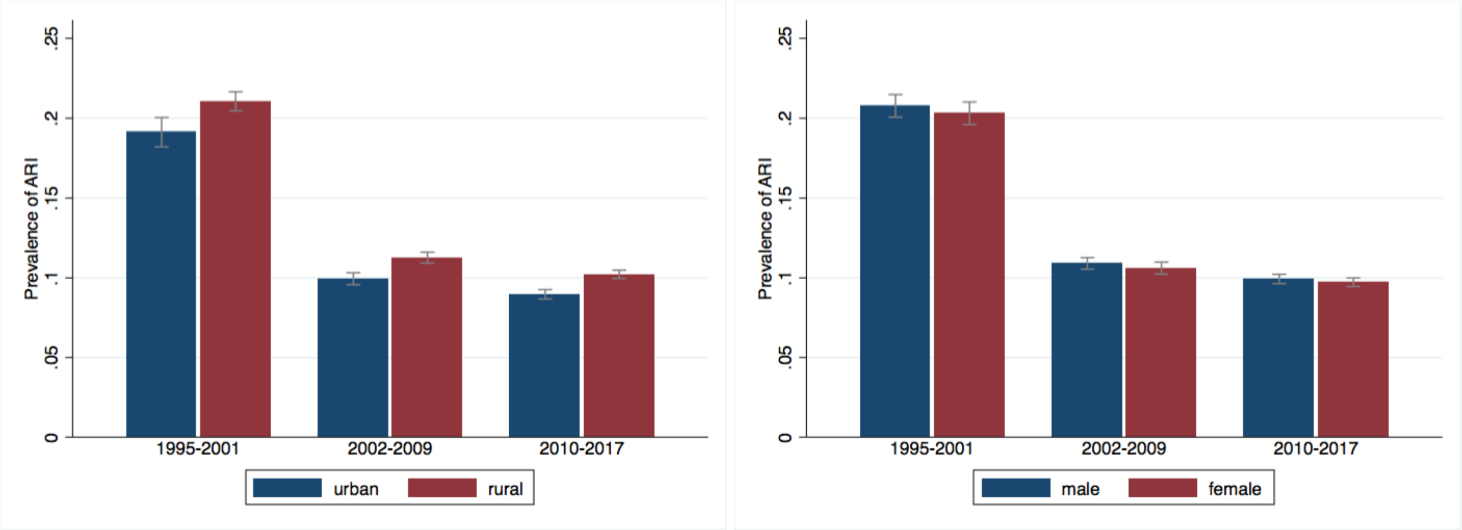

Figure S3. Prevalence of fever over time, disaggregated by location and gender

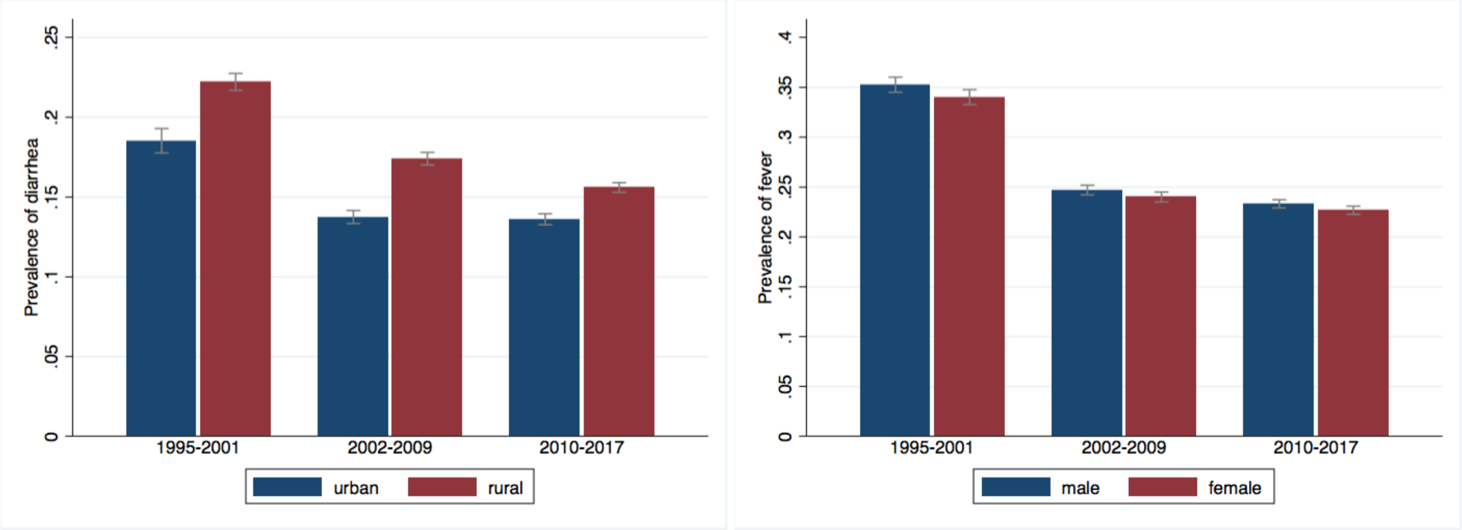

Figure S4. Prevalence of malaria over time, disaggregated by location and gender

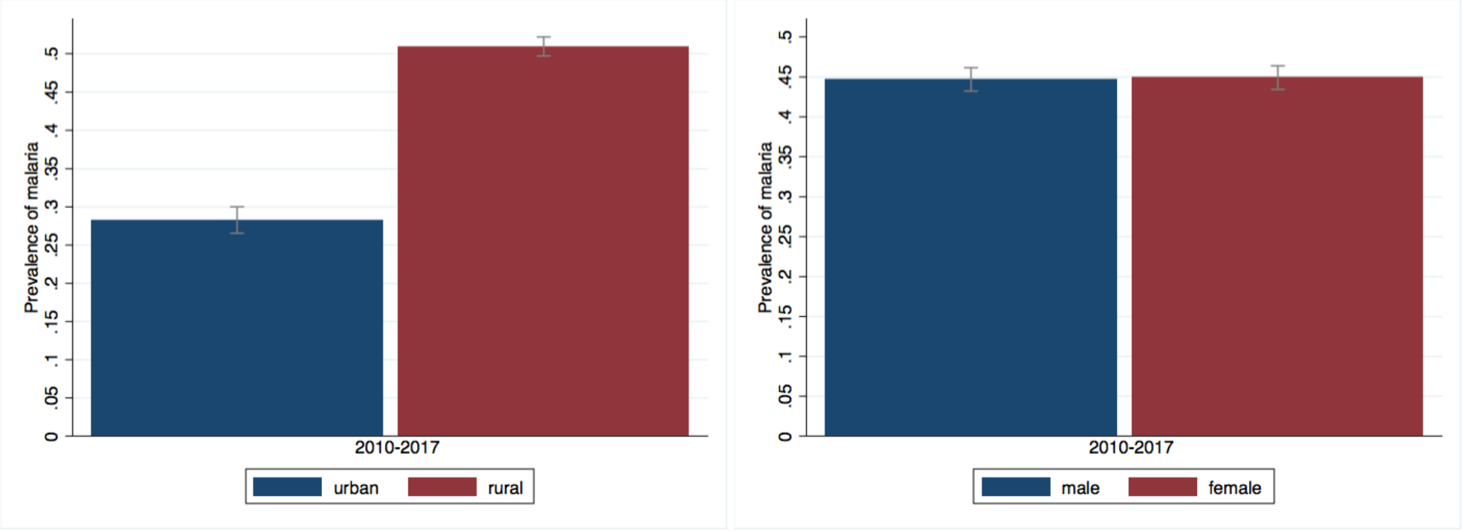

Figures S5 – S7. Prevalence of childhood diseases over time for all countries

Figure S5. Prevalence of diarrhoea over time by country

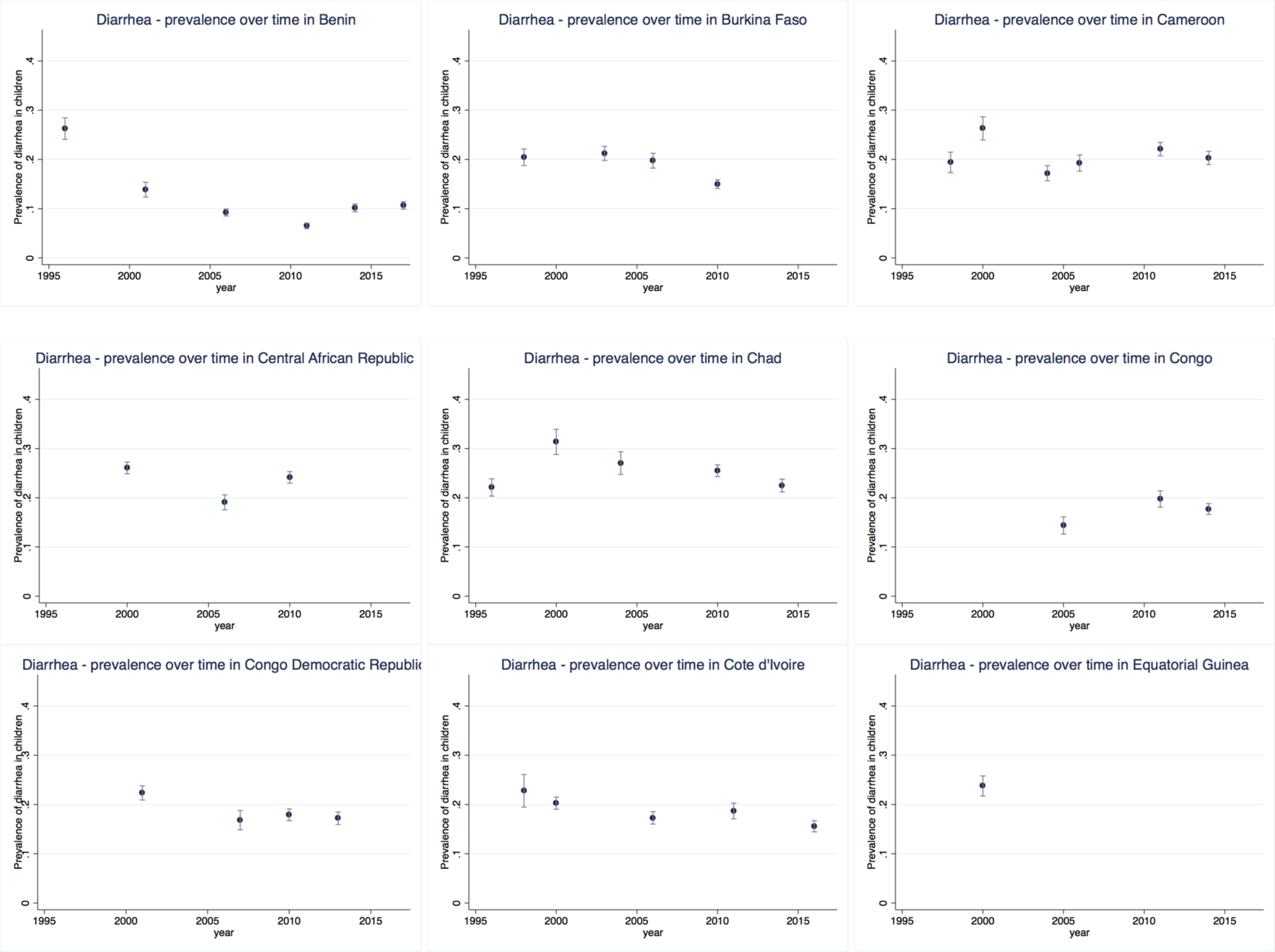

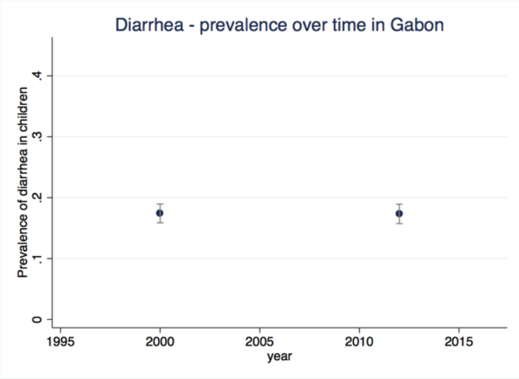
Diarrhea - prevalence over time in Gabon
Prevalence of diarrhea in children
0
.1
.2
.3
.4
1995
2000
2005
2010
2015
year

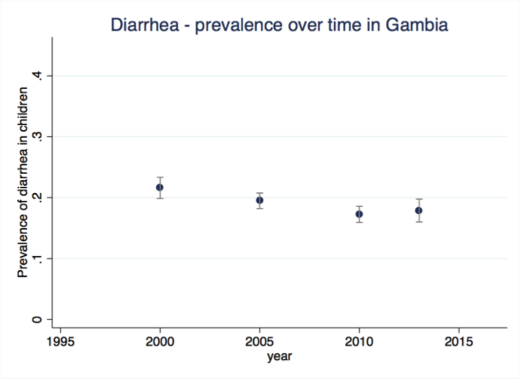
Diarrhea - prevalence over time in Gambia
Prevalence of diarrhea in children
0
.1
.2
.3
.4
1995
2000
2005
2010
2015
year

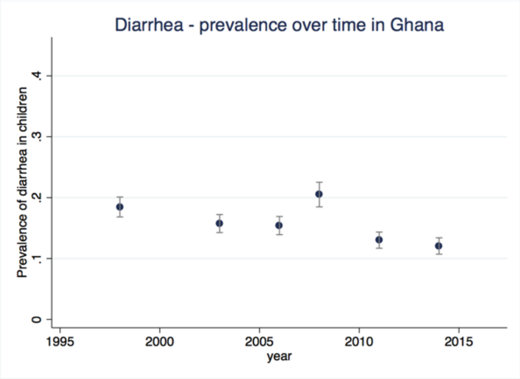
Diarrhea - prevalence over time in Ghana
Prevalence of diarrhea in children
0
.1
.2
.3
.4
1995
2000
2005
2010
2015
year

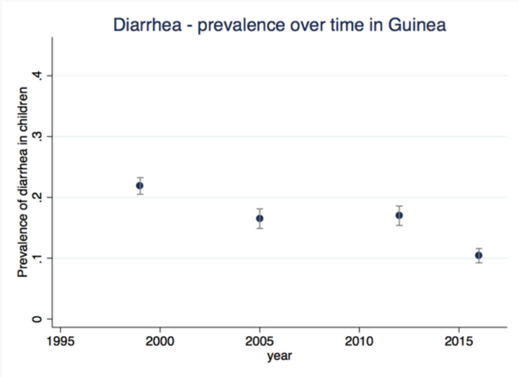
Diarrhea - prevalence over time in Guinea
Prevalence of diarrhea in children
0
.1
.2
.3
.4
1995
2000
2005
2010
2015
year

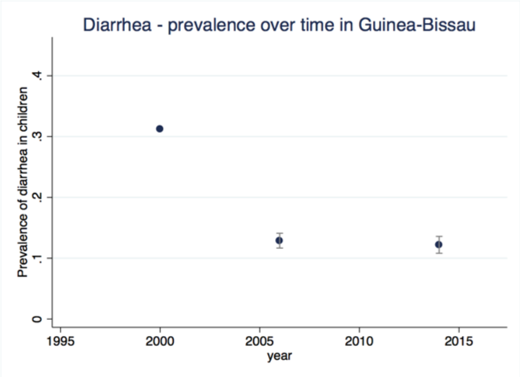
Diarrhea - prevalence over time in Guinea-Bissau
Prevalence of diarrhea in children
0
.1
.2
.3
.4
1995
2000
2005
2010
2015
year

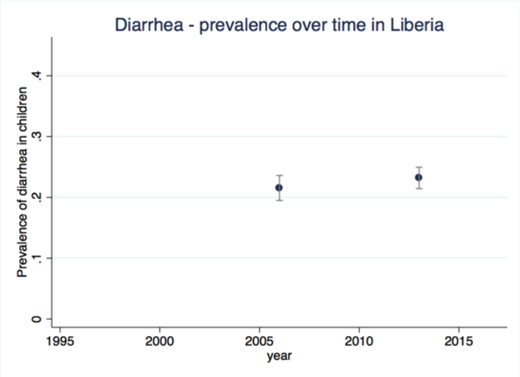
Diarrhea - prevalence over time in Liberia
Prevalence of diarrhea in children
0
.1
.2
.3
.4
1995
2000
2005
2010
2015
year

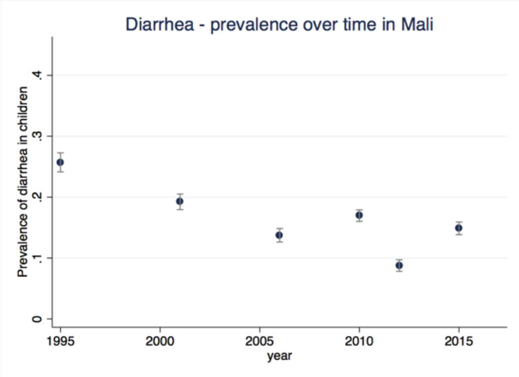
Diarrhea - prevalence over time in Mali
Prevalence of diarrhea in children
0
.1
.2
.3
.4
1995
2000
2005
2010
2015
year

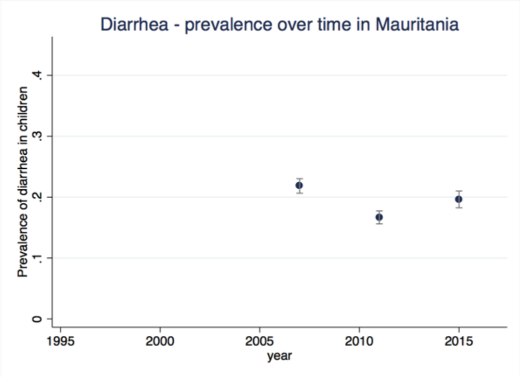
Diarrhea - prevalence over time in Mauritania
Prevalence of diarrhea in children
0
.1
.2
.3
.4
1995
2000
2005
2010
2015
year

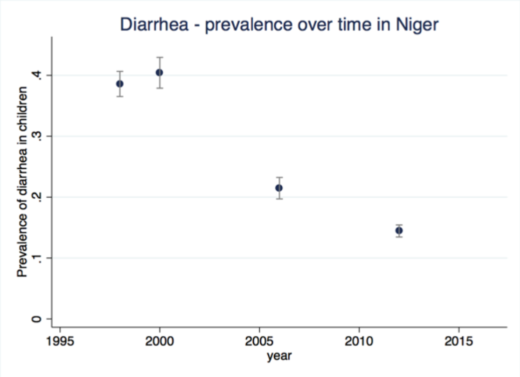
Diarrhea - prevalence over time in Niger
Prevalence of diarrhea in children
0
.1
.2
.3
.4
1995
2000
2005
2010
2015
year

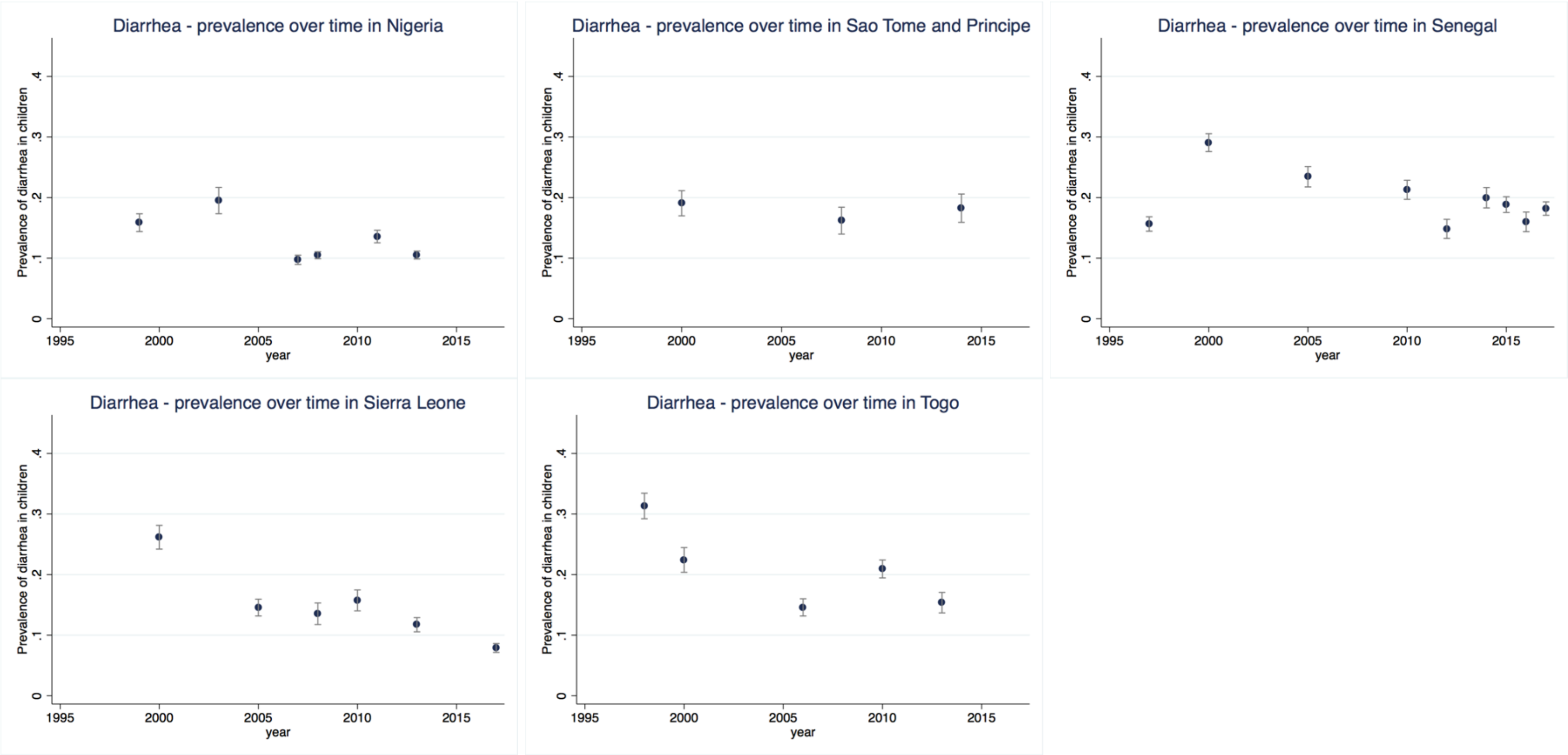

Figure S6. Prevalence of acute respiratory infections over time by country.

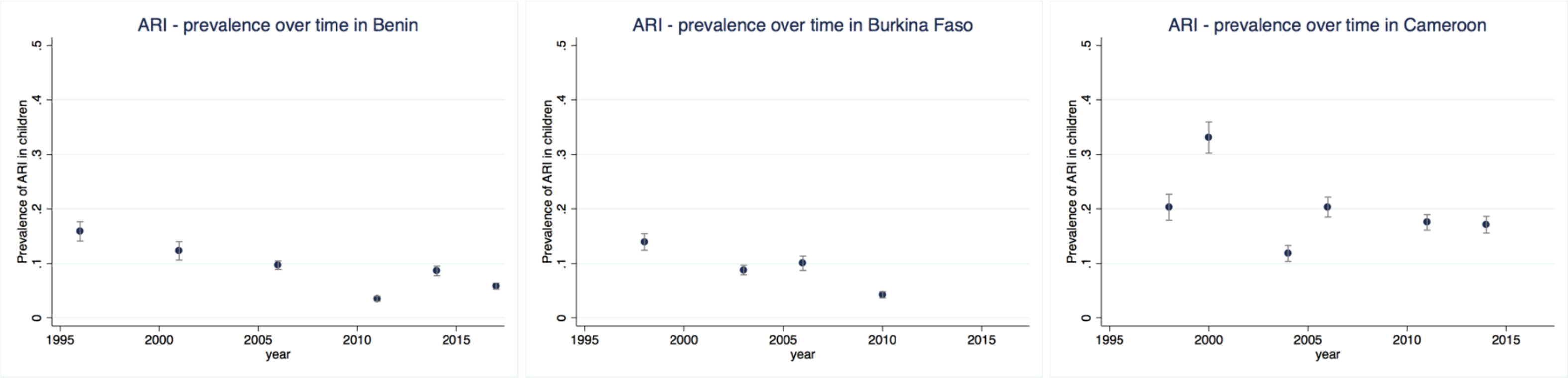

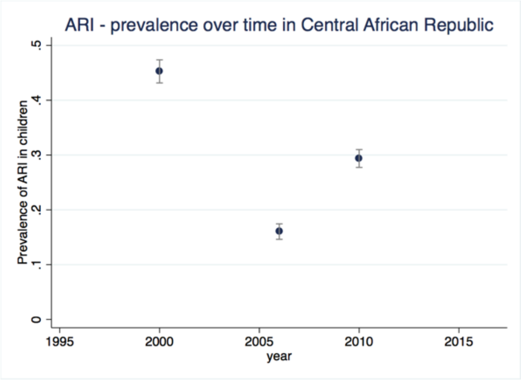
ARI - prevalence over time in Central African Republic
Prevalence of ARI in children
0
.1
.2
.3
.4
.5
1995
2000
2005
2010
2015
year

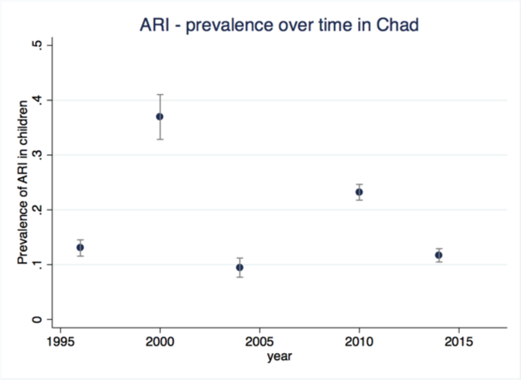
ARI - prevalence over time in Chad
Prevalence of ARI in children
0
.1
.2
.3
.4
.5
1995
2000
2005
2010
2015
year

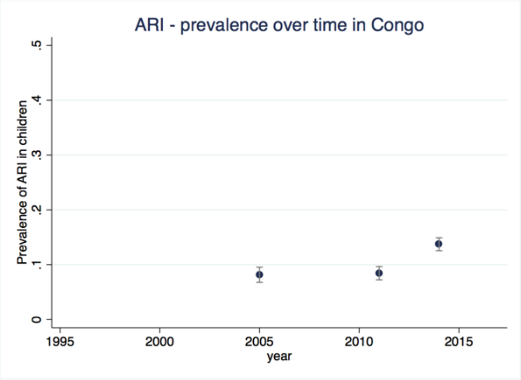
ARI - prevalence over time in Congo
Prevalence of ARI in children
0
.1
.2
.3
.4
.5
1995
2000
2005
2010
2015
year

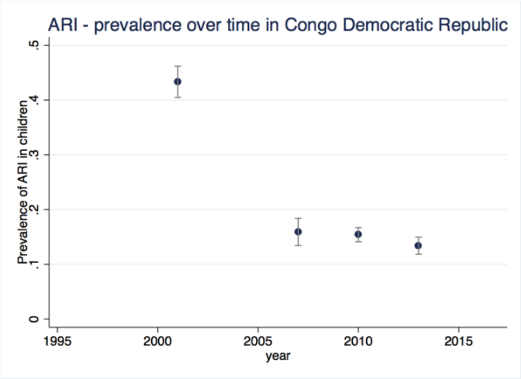
ARI - prevalence over time in Congo Democratic Republic
Prevalence of ARI in children
0
.1
.2
.3
.4
.5
1995
2000
2005
2010
2015
year

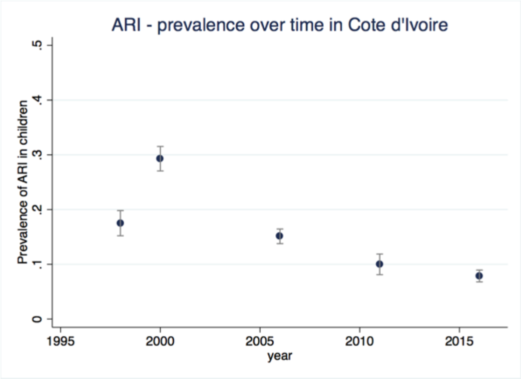
ARI - prevalence over time in Cote d'Ivoire
Prevalence of ARI in children
0
.1
.2
.3
.4
.5
1995
2000
2005
2010
2015
year

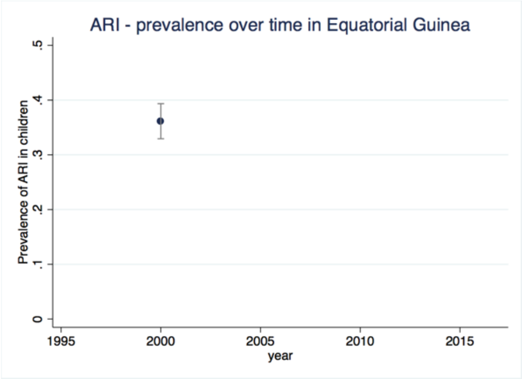
ARI - prevalence over time in Equatorial Guinea
Prevalence of ARI in children
0
.1
.2
.3
.4
.5
1995
2000
2005
2010
2015
year

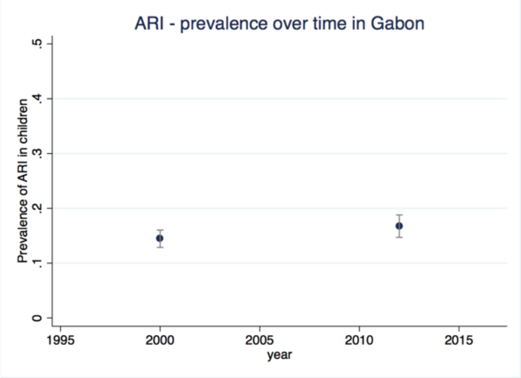
ARI - prevalence over time in Gabon
Prevalence of ARI in children
0
.1
.2
.3
.4
.5
1995
2000
2005
2010
2015
year

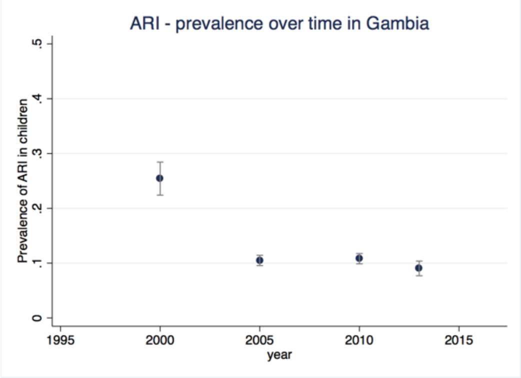
ARI - prevalence over time in Gambia
Prevalence of ARI in children
0
.1
.2
.3
.4
.5
1995
2000
2005
2010
2015
year

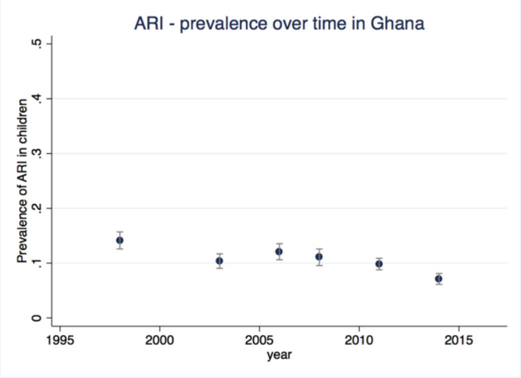
ARI - prevalence over time in Ghana
Prevalence of ARI in children
0
.1
.2
.3
.4
.5
1995
2000
2005
2010
2015
year

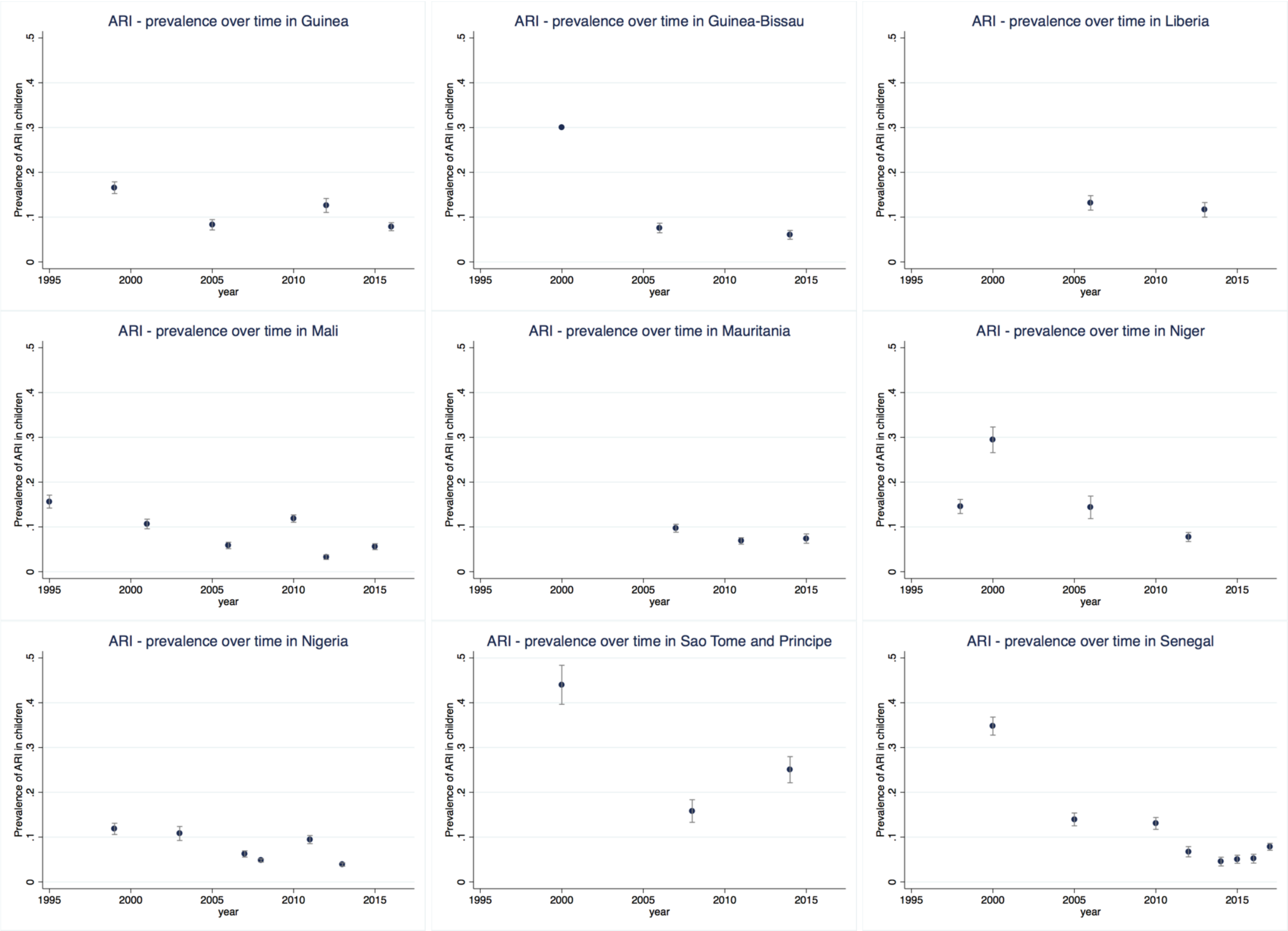
ARI - prevalence over time in Guinea
ARI - prevalence over time in Guinea-Bissau
ARI - prevalence over time in Liberia
ARI - prevalence over time in Mali
ARI - prevalence over time in Mauritania
ARI - prevalence over time in Niger
ARI - prevalence over time in Nigeria
ARI - prevalence over time in Sao Tome and Principe
ARI - prevalence over time in Senegal
Prevalence of ARI in children
0
.1
.2
.3
.4
.5
year
1995
2000
2005
2010
2015

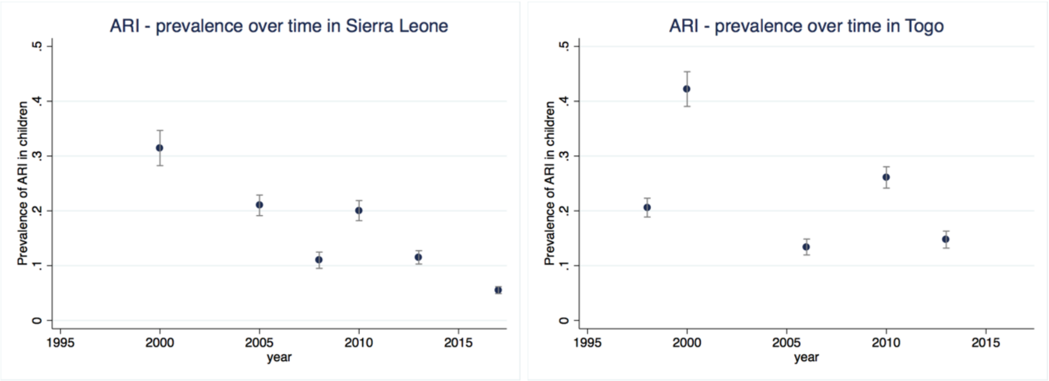

Figure S7. Prevalence of fever over time by country.

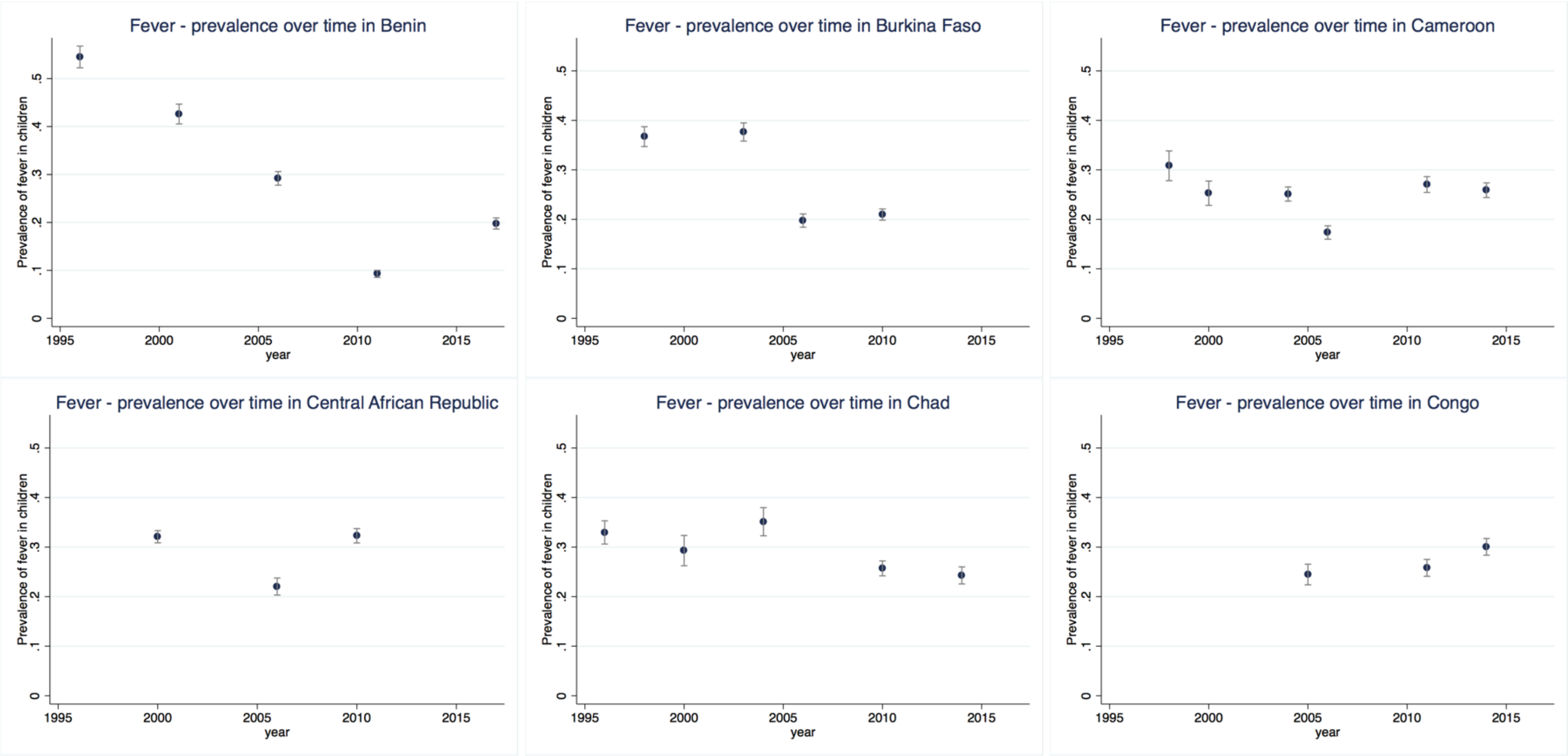

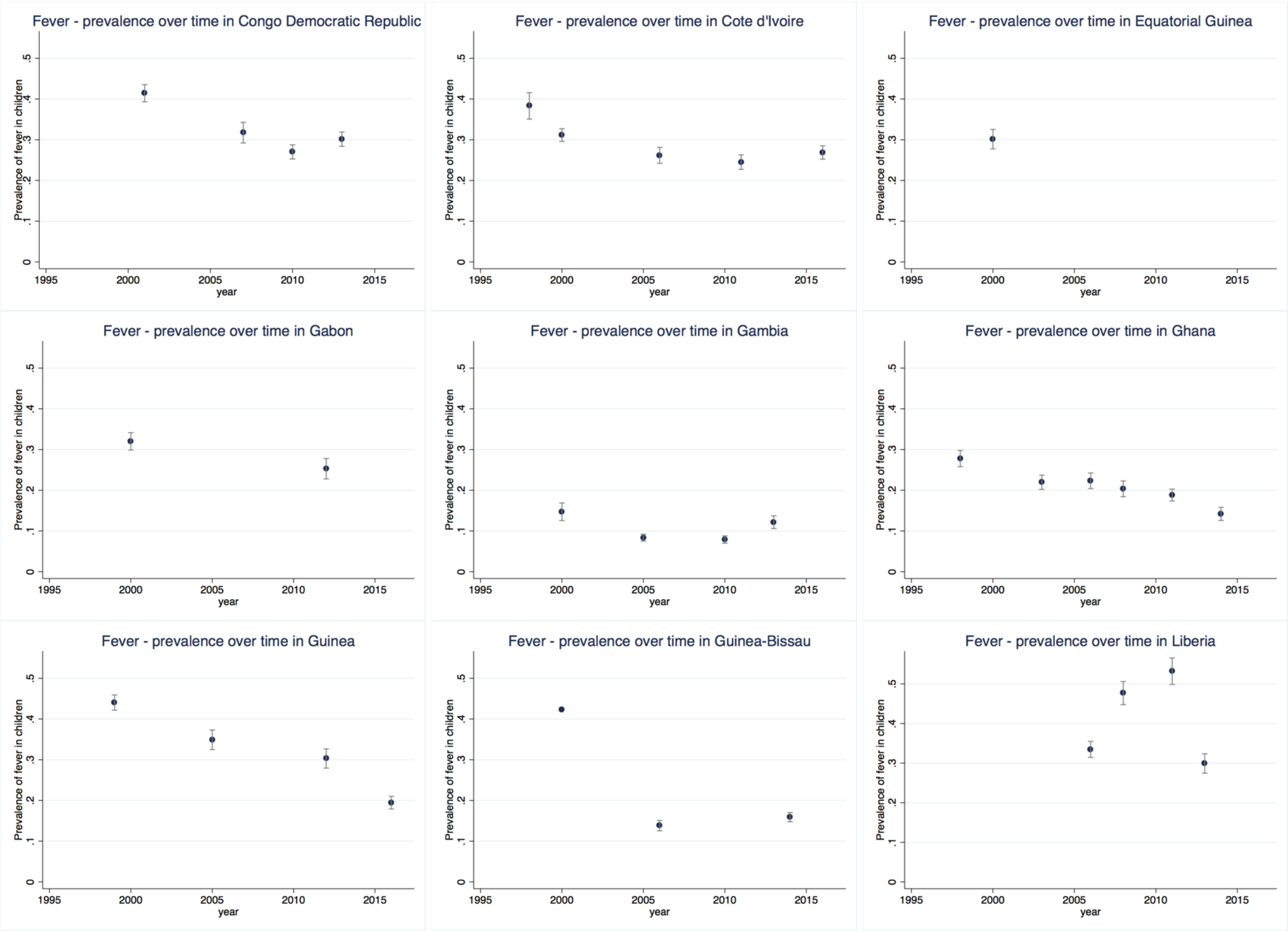
Fever - prevalence over time in Congo Democratic Republic
Fever - prevalence over time in Cote d'Ivoire
Fever - prevalence over time in Equatorial Guinea
Fever - prevalence over time in Gabon
Fever - prevalence over time in Gambia
Fever - prevalence over time in Ghana
Fever - prevalence over time in Guinea
Fever - prevalence over time in Guinea-Bissau
Fever - prevalence over time in Liberia
Prevalence of fever in children
0
.1
.2
.3
.4
.5
1995
2000
2005
2010
2015
year

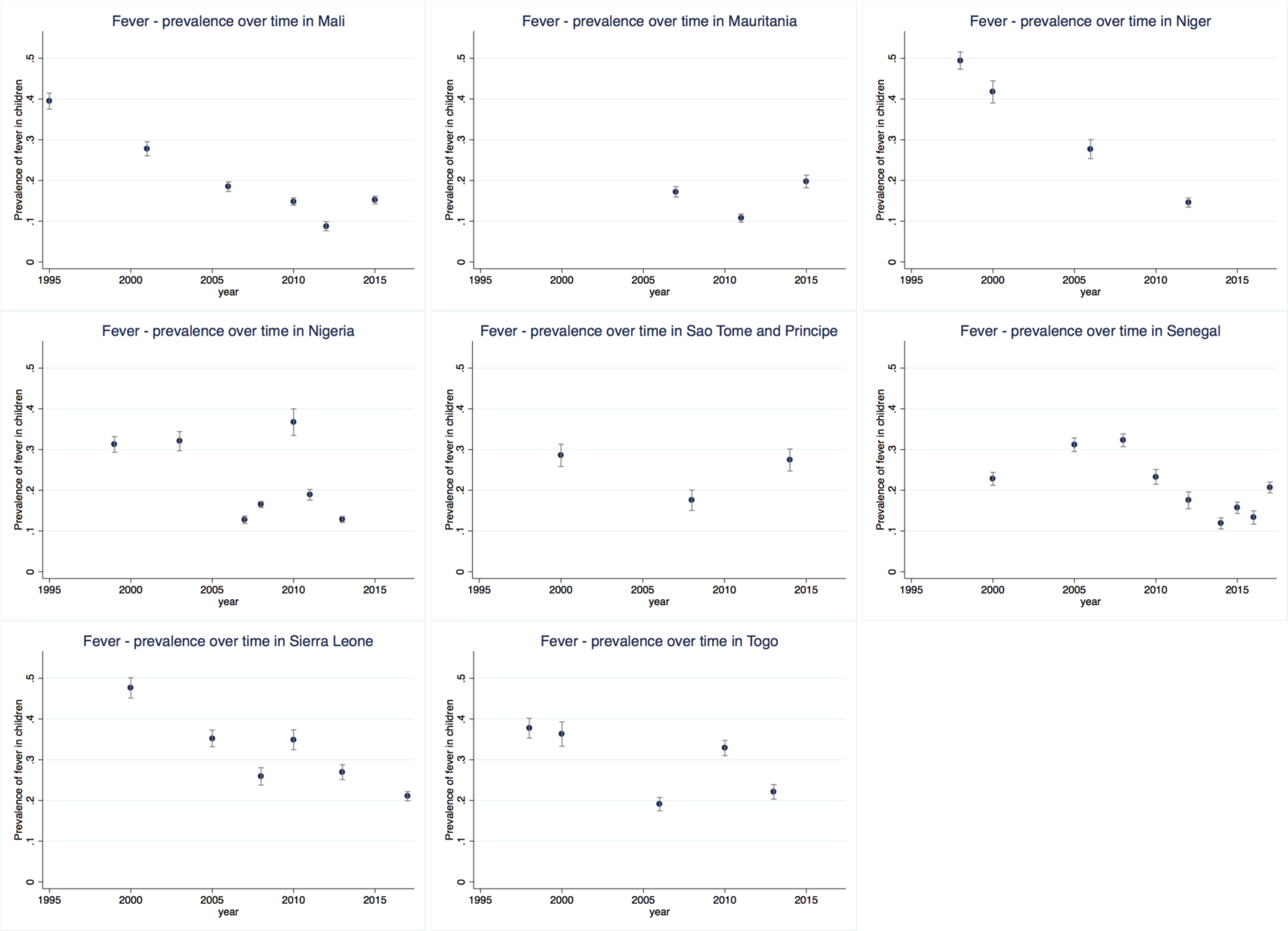
Fever - prevalence over time in Mali
Fever - prevalence over time in Mauritania
Fever - prevalence over time in Niger
Fever - prevalence over time in Nigeria
Fever - prevalence over time in Sao Tome and Principe
Fever - prevalence over time in Senegal
Fever - prevalence over time in Sierra Leone
Fever - prevalence over time in Togo
Prevalence of fever in children
0
.1
.2
.3
.4
.5
year
1995
2000
2005
2010
2015
